# Anti-VEGF therapy for the long-term management of diabetic macular edema: a treat-to-target strategy based on macular morphology

**DOI:** 10.1007/s00417-024-06558-y

**Published:** 2024-07-12

**Authors:** Shintaro Nakao, Sentaro Kusuhara, Tomoaki Murakami

**Affiliations:** 1 Department of Ophthalmology, Juntendo University Graduate School of Medicine, Tokyo, Japan; 2 Division of Ophthalmology, Department of Surgery, Kobe University Graduate School of Medicine, Kobe, Japan; 3 Department of Ophthalmology and Visual Sciences, Kyoto University Graduate School of Medicine, Kyoto, Japan

**Keywords:** Anti-VEGF therapy, Diabetic macular edema, Retinal fluid, Retinal thickness, Treat-to-target, Visual acuity

## Abstract

In an aging population, the prevalence and burden of diabetes mellitus, diabetic retinopathy, and vision-threatening diabetic macular edema (DME) are only expected to rise around the world. Similarly to other complications of diabetes mellitus, DME requires long-term management. This article aims to review the current challenges associated with the long-term management of DME, opportunities to improve outcomes for patients, and to develop a treat-to-target strategy based on macular morphology. At present, intravitreal anti–vascular endothelial growth factor (VEGF) therapy is the standard of care for the management of DME; however, best-achievable vision outcomes with treatment are reliant on frequent injections and close monitoring, which are difficult to maintain in current clinical practice because of the burden this imposes on patients. Achieving and maintaining good vision with treatment are the most important factors for patients with DME. Landmark trials have shown that vision gains with anti-VEGF therapy are typically accompanied by anatomical improvements (e.g., reductions in retinal thickness); therefore, multimodal imaging measures of macular morphology are often used in patients with DME to guide real-world treatment decisions. We would like to propose a hypothetical treat-to-target algorithm to guide physicians on treatment strategies for the long-term management of DME. Alternative measures of retinal fluid (e.g., persistence, stability, location) may be stronger predictors of visual acuity in DME, although further research is required to confirm whether alternate quantifiable biomarkers such as subretinal fluid and intraretinal fluid volumes can be used as a biomarker of clinical improvement. Identifying novel biomarkers and treatments that target neuroinflammation and neurodegeneration, improving patient-physician communication around treatment adherence, and using treat-to-target measures may help to ensure that the long-term benefits of treatment are realized.

## Key messages

***What is known:***

• Intravitreal anti-vascular endothelial growth factor (VEGF) therapy is the standard of care for patients with diabetic macular edema (DME); however, the burden of frequent injections is a key reason why real-world vision outcomes are often inferior to those reported in clinical trials.

• There is increasing evidence that the persistence, stability, and location of retinal fluid may be stronger predictors of visual acuity in DME; and that achieving rapid, stable, and sustained fluid control with anti-VEGF treatment could be an important treatment goal with the aim of improving longer-term outcomes.

***What is new***:

• A hypothetical treat-to-target algorithm to guide physicians on treatment paths for the long-term management of DME was proposed.

• Introducing a treat-to-target strategy, which sets treatment targets based on factors such as intraretinal fluid volume, and involves selecting customized treatment regimens and conducting regular assessments, could improve the future management of DME.

## Introduction

Diabetes mellitus is a chronic metabolic disease that affects approximately 537 million adults (10.5%) worldwide [1]. The prevalence of diabetes increases with advancing age, and the challenges of managing older people further exacerbate the burden of diabetes on patients, caregivers, and healthcare systems [1–3]. With aging populations and increasing adoption of westernized lifestyles around the world, the global prevalence of diabetes is expected to rise to approximately 783 million adults (12.2%) by 2045, including 24.7% of people aged 75–79 years [1].

Diabetic retinopathy is a common microvascular complication of diabetes, and one of the leading causes of visual impairment and blindness among people aged 50 years and older [4]. Vision loss associated with diabetic retinopathy is often caused by diabetic macular edema (DME), characterized by increased retinal vascular permeability and fluid accumulation in the macula [5]. Intravitreal anti–vascular endothelial growth factor (VEGF) therapy is currently the mainstay of treatment for DME; however, vision outcomes reported in landmark anti-VEGF trials have been difficult to achieve and maintain in real-world clinical practice [6–10].

Given the extended life expectancy of individuals in aging societies, long-term DME management is necessary to ensure that patients can maintain their vision, independence, participation in society, physical and cognitive health, and quality of life (QoL) [11]. In particular, visual impairment in older individuals becomes problematic in terms of frailty. It can prove difficult to improve vision with treatment in older patients if the intervention is made too late, so in our view, early treatment of DME is necessary. However, optimizing treatment and vision outcomes for older patients has proven difficult in real-world settings, for reasons including comorbidities, geriatric syndrome, and socioeconomic status [12]. In recent years, new anti-VEGF drugs have been approved, expanding the range of treatment options, but randomized controlled trials (RCTs) have only demonstrated the efficacy of specific drug treatment regimens and have not attempted to determine which treatment strategies can optimize individual patient outcomes. Herein, we review the barriers to optimal anti-VEGF therapy in current clinical practice and discuss the utility of a treat-to-target approach using morphological indicators to guide treatment, in order to preserve vision over the long term in patients with DME.

## Current state of anti-VEGF therapy for the management of DME

Since the initial discovery that VEGF upregulation is a key driver of vascular leakage in an animal model of the diabetic retina [13], and subsequent confirmation of this in individuals with DME [14, 15], intravitreal anti-VEGF therapy has become the standard of care around the world [16–20]. This shift in the DME treatment paradigm was informed by several studies that demonstrated superior visual and anatomical improvements with anti-VEGF agents over existing laser therapies [21–24]. In August 2012, ranibizumab was the first anti-VEGF agent approved by the United States (US) Food and Drug Administration (FDA) for the treatment of DME, followed by aflibercept in July 2014, faricimab (dual VEGF-A/angiopoietin-2 inhibitor) in January 2022, brolucizumab in June 2022, and high-dose aflibercept in August 2023 [25]. In August 2022, ranibizumab-eqrn became the first FDA-approved anti-VEGF biosimilar for the treatment of DME [25]; several other biosimilars are approved or in development around the world [26], which will further expand the anti-VEGF treatment options available to patients and physicians.

Several real-world studies have found that the clinical benefits of anti-VEGF therapy for DME, as reported in landmark trials, have been difficult to replicate in clinical practice [6–10]. Table 1 summarizes the broad differences in results between RCTs and real-world data. In RCTs, mean best-corrected visual acuity (BCVA) gains of 6–13 Early Treatment Diabetic Retinopathy Study (ETDRS) letters were achieved with approximately 7–12 intravitreal anti-VEGF injections during the first year of treatment [21, 22, 27–31]. In contrast, the prospective real-world MERCURY study found that, on average, patients in Japan received 3–4 anti-VEGF injections during the first year of treatment, and consequently achieved inferior vision gains of approximately 4 ETDRS letters [9]. During the second year of treatment, patients in MERCURY received an additional 1–2 anti-VEGF injections on average, and achieved small additional vision gains of approximately 2.5 ETDRS letters [10].

**Table 1:** Generalized differences in study conduct and results between RCTs and real-world studies of anti-VEGF treatments for DME, based on the expert opinion of the authors

	RCTs	Real-world studies
Assessments/setting	Drug efficacy and safety (in an ideal environment)	Drug effectiveness and safety (in clinical practice)
Number of treatments investigated	Many	Less
Improvement in vision	Good	Insufficient
Improvement in retinal thickness	Good	Inadequate?
Duration of treatment	Limited to study duration^a^	Varies by individual
Lost to follow-up	During the trial	Individual differences
Other	Single-treatment regimens	Combination of various treatments?

^a^For example, the DRCT.net Protocol T study followed up patients for up to 5 years (Glassman 2020).

*DME*, diabetic macular edema; *DRCR*.net, Diabetic Retinopathy Clinical Research Network; *RCTs*, randomized controlled trials; *VEGF*, vascular endothelial growth factor

Data from the US and Europe have shown that patients with DME are significantly more likely to cancel, or simply not attend, scheduled retina specialist appointments versus those with neovascular age-related macular degeneration (nAMD) [32]. Adherence to appointments may be particularly difficult for patients with DME, who are likely to be of working age [19], and who often have more medical appointments overall to address other complications of their diabetes than diabetic patients without DME [32]. Furthermore, a survey of Japanese retina specialists that sought to characterize real-world anti-VEGF treatment practices for DME found that the most common barriers to ongoing anti-VEGF therapy were the high costs of treatment (86% of respondents) and the need for frequent injections (24% of respondents) [33]. Similarly in the American Society of Retina Specialists (ASRS) 2023 Global Trends in Retina survey, specialists from around the world commonly identified “frequent loss to follow-up” and “limitations of patient access to retina care” as the greatest socioeconomic challenges faced when treating patients with DME [34].

The burden of frequent appointments and anti-VEGF injections may be addressed through newer agents with extended durability (e.g., brolucizumab, faricimab), which have shown that robust vision gains and anatomical improvements can be maintained with dosing up to every 12–16 weeks [30, 31]. In addition, the increasing availability of anti-VEGF biosimilars may alleviate the financial burden of treatment on patients and healthcare systems [26]; however, increased physician-patient communication and education may be required to overcome the potential reluctance of patients to accept treatment with a generic agent [35]. A growing number of anti-VEGF treatment options for DME may also confound clinical decision-making, thus highlighting the need to differentiate between individual anti-VEGF agents and identify patient populations most likely to benefit from each treatment.

Future research directions in anti-VEGF therapy for DME include extended-release intraocular devices and gene therapy. The Port Delivery System with ranibizumab (PDS) is a surgical ocular implant that provides continuous ranibizumab therapy into the eye [36]. In the phase 3 Archway trial of 418 patients with nAMD, the PDS (refilled every 24 weeks) had similar efficacy to monthly ranibizumab injections [36]. A phase 3 trial of the PDS in DME is currently ongoing (NCT04108156). Two gene therapy products (RGX-314 and 4D-150), that are administered as a one-time injection and allow endogenous expression of anti-VEGF, are currently undergoing phase 2 trials in patients with DME (NCT04567550 and NCT05930561), with estimated primary completion dates in 2024.

### Anti-VEGF therapy for DME based on macular morphology

In addition to functional outcomes (i.e., visual acuity), clinical trials have consistently demonstrated the efficacy of anti-VEGF therapy using anatomical endpoints, including optical coherence tomography (OCT) measures of retinal thickness (e.g., central retinal thickness [CRT], central subfield thickness [CST]) and retinal fluid (e.g., subretinal fluid [SRF], intraretinal fluid [IRF]) [21, 22, 27–31, 37]. However, unlike vision gains, which are generally comparable across individual anti-VEGF therapies, comparative studies and meta-analyses have shown that anatomical responses to treatment can differ between agents [24, 29–31]. For example, a recent Cochrane review found no clinically important differences in 24-month BCVA gains between current anti-VEGF therapies for DME, but estimated that 24-month CRT reductions tended to favor brolucizumab and aflibercept over ranibizumab, bevacizumab, and ranibizumab plus prompt or deferred laser therapy [24].

Given that retinal thickness and fluid are important features of DME, international guidelines and clinical trials frequently use anatomical measures to monitor anti-VEGF treatment response and guide retreatment decisions. Current clinical guidelines recommend the use of OCT, in conjunction with fundus photography and fluorescein angiography, to diagnose DME based on morphological indicators, and advocate that patients can be monitored and assessed for anti-VEGF retreatment based on visual acuity and OCT findings [16–18, 20]. Similarly in clinical trials, OCT-based anatomical criteria are routinely used to select patients for study enrolment, and to determine anti-VEGF dosing intervals in personalized treatment regimens (e.g., pro re nata [PRN; as-needed], treat-and-extend) [21, 27, 29–31].

**Fig. 1:**
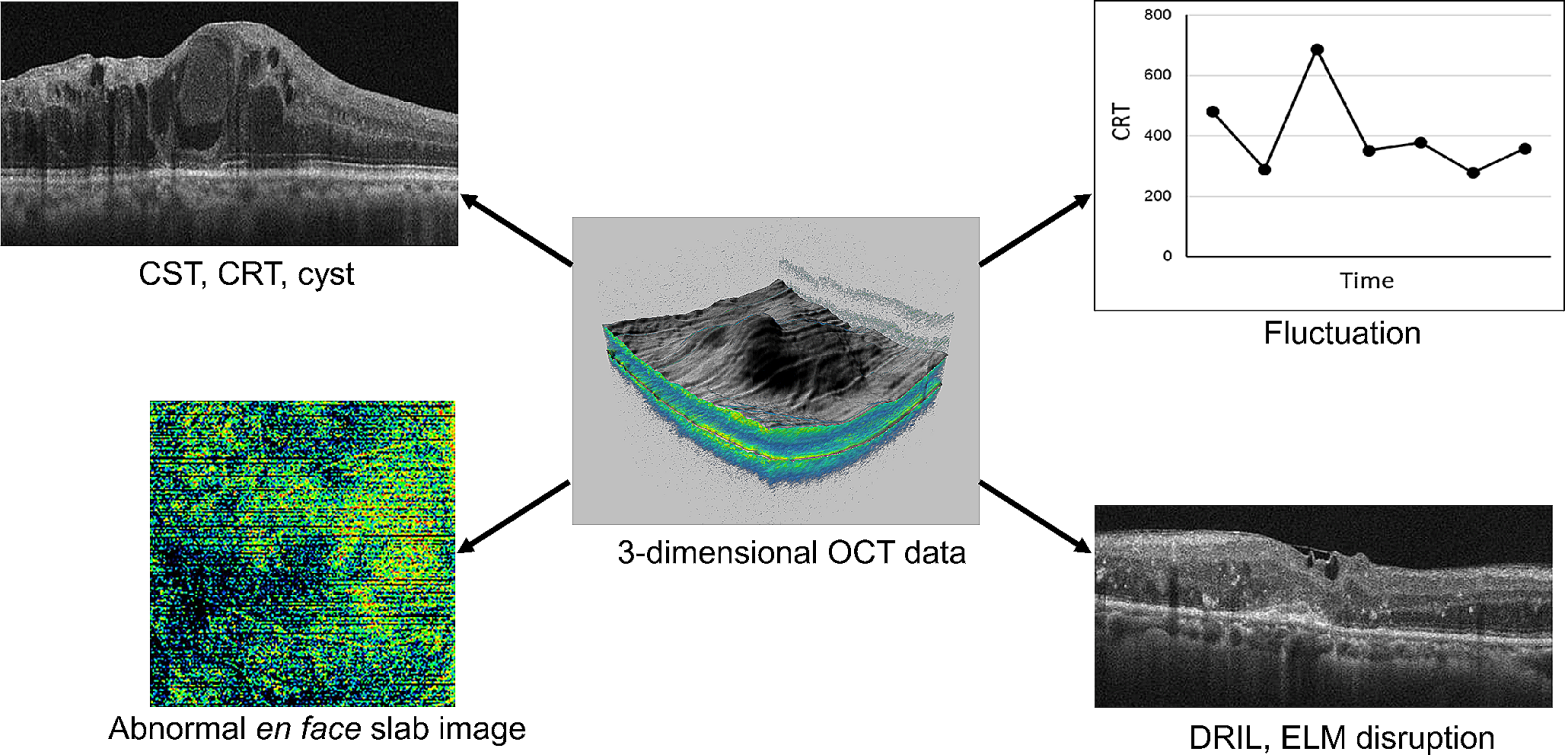
Representative examples of typical 3-D optical coherence tomography (OCT) parameters assessed in patients with diabetic macular edema (DME) in clinical practice. *CRT* central retinal thickness

CST, central subfield thickness; DRIL, disorganization of retinal inner layers; ELM, external limiting membrane.

In line with clinical guidelines and trial protocols, OCT assessments of retinal morphology are widely used to guide the management of DME in current clinical practice [19, 33, 38]. Figure 1 illustrates typical OCT parameters assessed in everyday clinical practice; however, there is no universally agreed-upon approach for utilizing these parameters in a clinical context. A survey found that 68% of retina specialists agreed that OCT findings (particularly CRT and OCT retinal maps) are the most important considerations when initiating treatment for DME, followed by visual acuity (23% of respondents) and subjective symptoms (8% of respondents) [38]. In comparison, a similar cross-sectional study of ophthalmologists found that visual acuity was the most useful assessment to guide DME treatment initiation (44% of respondents) followed by OCT (31% of respondents) [33].

Despite the routine use of anatomical measures in clinical trials and clinical practice, no consensus has been reached on how OCT findings should be interpreted and used to inform DME treatment decisions [39]. For example, there is no consistent CRT threshold to identify the presence or absence of DME, nor consistent definition of “stable” CRT to guide anti-VEGF retreatment.

### Relationship between macular morphology and visual acuity in DME

Although clinical trials have shown that vision gains with anti-VEGF therapy are typically accompanied by reductions in retinal thickness, several studies have found that the correlation between these two endpoints is modest at best. For example, analyses of the Diabetic Retinopathy Clinical Research Network (DRCR.net) Protocol A and Protocol T trials aimed to characterize the relationships between retinal thickness and visual acuity, and between changes in these parameters after treatment, in patients with DME [40, 41]. Both studies estimated small-to-moderate correlations between retinal thickness and visual acuity before and after treatment, suggesting that measuring retinal thickness at a single time point, or as the change between two time points, may not be reliable surrogate markers for vision outcomes [40, 41].

However, recent studies exploring other measures of retinal fluid have progressively revealed associations between morphological indicators and visual acuity in DME. In a post hoc analysis of the DRCR.net Protocol I study, the duration and amount of residual edema after anti-VEGF treatment with ranibizumab were each significantly and negatively correlated with longer-term vision outcomes [42]. On average, smaller vision gains over 3 years of follow-up were observed among patients with more persistent residual fluid during the first year of treatment (based on the number of study visits with CRT ≥ 250 μm), and among those with higher levels of edema during the same period (based on the amount by which CRT exceeded 250 μm) [42]. Moreover, a post hoc analysis of DRCR.net Protocols T and V found that larger fluctuations in CST (i.e., less stable fluid control) during 1 year of anti-VEGF therapy, focal/grid laser treatment, or observation were associated with worse vision outcomes over 2 years [43]. When SRF and IRF volumes were quantified separately, another analysis of Protocol T data showed that for every 10-nL reduction of central IRF and SRF achieved with anti-VEGF treatment, BCVA was significantly improved by 0.15 and 0.34 ETDRS letters, respectively (both *P* < 0.001) [37]. In a subsequent retrospective cohort study that divided IRF into compartments of the inner nuclear layer (INL) and outer plexiform layer (OPL), fluid volume in the INL demonstrated a stronger correlation with visual acuity than fluid volume in the OPL, whole macular fluid volume, and CST [44]. Taken together, these data suggest that the persistence, stability, and location of retinal fluid may be stronger predictors of visual acuity than retinal thickness in patients with DME; and that treatments providing rapid, stable, and sustained fluid control may improve long-term vision outcomes.

### Strategies to optimize long-term anti-VEGF therapy for DME

Although retina specialists believe that high treatment costs and frequent injections are common barriers to ongoing anti-VEGF therapy in clinical practice [33], patient surveys suggest that vision is the most important consideration for those with DME, and that many patients would be willing to accept increased treatment burden in exchange for better vision outcomes [45, 46]. For example, in a US survey of patients receiving anti-VEGF therapy for DME or nAMD, achieving good vision was the most important factor in treatment decisions (40% relative importance), followed by low cost to the patient (23%), on-label drug status (21%), less frequent treatment intervals (12%), and low cost to the insurance provider (3%) [45]. Another survey from the US by Mason and colleagues further highlighted the importance of vision in patients with DME: 83% of respondents indicated that they would accept 15–16 intravitreal injections to gain 2 lines of Snellen visual acuity, 91% would sacrifice zero lines of vision in order to receive fewer treatments, and 76% were willing to attend 12 treatment visits per year in order to maintain their vision [46]. The willingness to accept increased treatment burden may have been influenced in these US surveys by patient health insurance status and level of affluence. In the survey by Mason and colleagues, who collected sociodemographic and healthcare insurance status data, laser treatment was preferred over injections in unemployed respondents compared with employed respondents [46]. The authors of the latter study concluded that patient demographics influenced their responses regarding their preferences for DME treatment [46].

Nevertheless, patients may be more willing to accept an increased treatment burden when they have a clear understanding of treatment requirements and expected outcomes; however, physician-patient communication around optimal diabetes management may be lacking in current clinical practice. In a global survey of physicians involved in the early treatment of patients with type 2 diabetes, most respondents (88%) agreed that conversations at the time of diagnosis can meaningfully impact a patient’s acceptance and self-management of their condition over time [47]. Despite this, almost all physicians (99%) reported communication challenges when discussing type 2 diabetes management with patients; these included their sense of disappointment with patient attitudes (e.g., difficulty adhering to treatment recommendations, failure to understand the seriousness of the condition), and their sense of frustration with the clinical setting (e.g., insufficient time or resources to support patients, difficulty responding to emotional responses from patients) [47]. In our view, the stigma of diabetes a patient may experience, which is well documented in adolescents and young adults with diabetes (especially in female patients and/or patients with elevated glycated hemoglobin levels or diabetic retinopathy) [48], may hinder patient-physician communication. It is possible that if the clinician discusses diabetes stigma with the patient as part of providing comprehensive diabetes care, overall patient-physician communication may be improved.

For patients with DME, strategies to improve physician-patient communication are needed to ensure that the long-term benefits of anti-VEGF therapy are realized. Early discussions should aim to educate patients that best-achievable responses to anti-VEGF therapy require frequent injections and close monitoring, and that DME is a chronic disease that requires lifelong management. Patients may also be encouraged by 5-year data from the DRCR.net Protocol I trial, which found that many patients were able to reach a state of “remission”, where they could maintain initial vision outcomes with very few additional anti-VEGF injections over time [49].

Patients should also be made aware that DME is a heterogeneous disease, and while some may be able to achieve vision gains with anti-VEGF therapy, a more realistic treatment goal for most patients is to delay disease progression and avoid further vision loss. This could be illustrated with OCT images showing that retinal thickness and fluid are improved with treatment, and an explanation of increasing evidence that morphological indicators are related to longer-term vision outcomes. By demonstrating that anti-VEGF therapy has an observable effect on retinal morphology and reevaluating treatment goals, patients may be motivated to continue anti-VEGF treatment and maintain long-term DME control.

### Treat-to-target: the future of life-long DME management?

In current clinical practice, personalized anti-VEGF regimens such as PRN (i.e., as-needed injections based on disease activity at regular visits) and treat-and-extend (i.e., intervals between injection visits are based on disease activity at the last visit) are often adopted to reduce the burden of treatment on patients with DME [50]. However, PRN regimens rely on frequent monitoring which is nevertheless burdensome and suboptimal in clinical practice, and treat-and-extend regimens are associated with more frequent injections versus PRN regimens [50].

To better facilitate long-term DME management, a personalized strategy may be treat-to-target which is widely used in many chronic diseases including rheumatoid arthritis, hypertension, diabetes, and osteoporosis [51–63], but is not well-studied in the setting of DME. Treat-to-target is not a treatment protocol but a treatment strategy, unlike PRN or treat-and-extend. Treat-to-target is not an alternative to PRN or treat-and-extend, but rather, it is a complementary strategy to improve/optimize personalized dosing like PRN and treat-and-extend regimens [50], and change pharmacological or non-pharmacological treatments if required. Key points in managing DME using a treat-to-target strategy, which considers established strategies in rheumatoid arthritis care, are shown in Table 2 [63].

**Table 2:** Key considerations in managing diabetic macular edema using a treat-to-target (T2T) strategy: lessons from rheumatoid arthritis care [63]

Basic T2T concepts in RA	Potential T2T concepts in DME
A. Treatment of RA should be based on agreement between the patient and rheumatologist	A. Treatment of DME should be based on agreement between the patient and retina specialist
B. Key treatment goals are to maximize long-term QoL by controlling symptoms, suppressing joint damage, and normalizing physical function and social participation	B. Key treatment goals are to maximize long-term QoL by improving QoV and anatomical outcomes, controlling retinal edema, reducing injection burden, and normalizing social participation
C. Decreasing inflammation is most important for achieving treatment goals	C. Reducing CRT is important for achieving treatment goals
D. T2T based on the assessment of disease activity and optimization of treatment to achieve goals is effective for improving outcomes	D. T2T based on the assessment of OCT and visual acuity and optimization of treatment to achieve goals is effective for improving outcomes

*CRT* central retinal thickness, *DME* diabetic macular edema, *OCT* optical coherence tomography, *QoL* quality of life, *QoV* quality of vision, *RA* rheumatoid arthritis

**Fig. 2:**
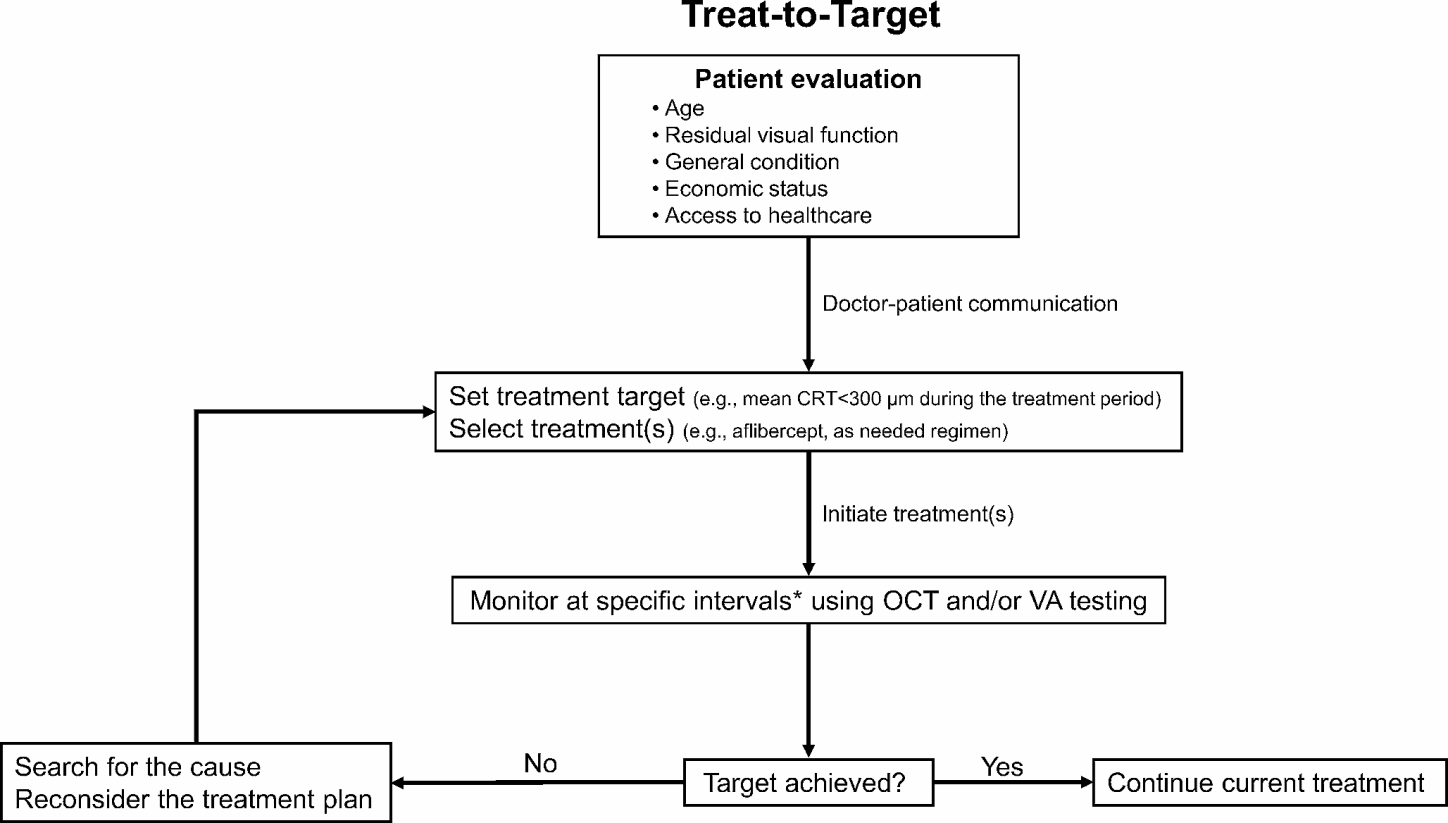
Hypothetical treat-to-target algorithm for the long-term management of diabetic macular edema. *Varies depending on whether it is a short-term target or a medium-to-long-term target. *CRT* central retinal thickness, *OCT* optical coherence tomography, *VA* visual acuity

An international task force has provided recommendations for treat-to-target in rheumatoid arthritis [63]. The primary treatment aim in rheumatoid arthritis is remission, with low disease activity being an alternative target in patients with long-established disease [63]. Follow-up every 1–3 months during active disease is recommended, with therapy changes as needed to meet treatment goals within 3–6 months [63]. Follow-up examinations should use composite measures of disease activity which include joint counts [63]. In rheumatoid arthritis, psoriatic arthritis, inflammatory bowel disease, and gout, using a treat-to-target strategy improves patient outcomes by controlling inflammation and reducing disability and structural damage [51, 52, 54, 56, 57, 59, 62, 63]. In hypertension and diabetes, such a strategy prevents end organ damage, disability, and premature mortality [58, 60]. In patients with osteoporosis and a history of fragility fracture, a treat-to-target approach may restore daily physical functioning and reduce subsequent fracture risk [61]. Despite these benefits, implementation of treat-to-target in clinical practice seems to lag behind expected benefits [51, 52, 59]. This may be due to less-than-ideal patient adherence to the treatment regimen and that selection of the correct treatment target for each individual needs to be better defined and validated [51, 52, 59]. Figure 2 represents a hypothetical treat-to-target strategy for the long-term management of DME.

Regarding future possible studies of a treat-to-target strategy in DME, although it would be ideal to conduct a RCT, it is probably not feasible. A single-arm prospective trial would be more realistic. We suggest an approach used by Verstappen and colleagues [64], which investigated whether a treat-to-target strategy, according to a strictly predefined protocol determined by a computerised decision program, was more beneficial compared with a conventional treatment approach in patients with rheumatoid arthritis. However, it is not clear whether the current proposal is suitable in terms of patient evaluation items, frequency of evaluation, etc., and future evaluation is required to determine whether this treat-to-target algorithm is effective in the clinical setting of DME.

## Future research directions

A deeper understanding of the pathophysiology of DME and better biomarkers are needed to make treat-to-target more robust. Future management of DME may be improved by increased understanding of the causes of DME via neuroinflammatory, neurodegenerative, and ischemic mechanisms [65]. Although diabetic retinopathy and DME are traditionally considered to be microvascular complications with VEGF representing a key therapeutic target, there is increasing evidence that chronic hyperglycemia in diabetes may trigger intraretinal neuroinflammation, neuronal degeneration, and retinal ischemia that precede microvascular, anatomical, and functional changes [65]. Key cellular and molecular pathways involved in neuroinflammation, blood-retina barrier breakdown, and extra-retinal neoangiogenesis in diabetic retinopathy continue to be identified (as reviewed elsewhere previously) [66]. Further research is also required to better understand the complex relationship between these vascular and anatomical changes and retinal function in patients with diabetes, as illustrated in a study on retinal vessel permeability in patients with diabetic retinopathy [67]. This study showed that retinal vessel hyperpermeability had a negative effect on retinal sensitivity (a measure of visual function) in areas with retinal edema and ischemia, whereas it had a positive effect in preserving retinal function in areas of ischemia with no edema [67]. Whether these results are also applicable to patients with DME could not be confirmed since patients with center-involving macular edema were excluded from this study [67], and represents an area for additional investigation.

There is increased effort in identifying new intraocular and multimodal imaging biomarkers that may be used to detect retinopathy earlier, assess disease severity and risk of progression, and develop treatment strategies beyond VEGF inhibition [65, 68]. For example, neuronal degeneration has been observed in the innermost retinal layer of patients with diabetes by using novel *en face* slab OCT imaging in a study of 72 patients that included 22 patients (31%) with no clinical diabetic retinopathy [69]. Further, this novel imaging technique was able to detect deterioration in the innermost layer over time (follow up mean 4.6 years), as retinal neurodegeneration progressed [69]. Some authors view better understanding of retinal neurodegeneration as an important path to finding treatments beyond VEGF inhibition, and have identified the neurovascular unit as a putative therapeutic target, especially since retinal neurodegeneration may precede vascular changes [70].

Understanding DME as a neurovascular disease may present opportunities to personalize future treatment based on individual vascular, neuroinflammatory, and neurodegenerative phenotypes, and may signal the next paradigm shift since the advent of anti-VEGF therapy.

Further research is needed to establish appropriate anti-VEGF treatment targets in DME and to determine whether treating to a specific anatomical target would optimize long-term outcomes in DME. Studies are required to identify surrogate biomarkers that may be used in DME trials in the same way that, for example, intraocular pressure has been used as a surrogate endpoint in glaucoma trials [71]. Two quantifiable biomarkers, SRF and IRF, have the potential to be expanded upon as anti-VEGF treatment targets in DME [37].

## Conclusions

Maintaining visual function throughout an individual’s lifetime is crucial for independent living and better QoL in an aging society; however, current anti-VEGF therapy is difficult to implement as an effective life-long treatment for patients with DME because of the associated treatment burden. Novel treatment targets still need to be identified, possibly by exploiting the complexity of DME pathogenesis, which includes vascular and ischemic changes, and retinal neurodegeneration. Identifying novel biomarkers and treatment targets, and expanding on current quantifiable biomarkers such as SRF and IRF, will assist in providing the necessary treatment options and patient management measures that allow for personalized treat-to-target strategies to take center-place, along with patient-physician communication, in the long-term management of patients with DME.

## Data Availability

Data sharing is not applicable to this review article as no new data were created or analyzed in this study.
